# The impact of low input DNA on the reliability of DNA methylation as measured by the Illumina Infinium MethylationEPIC BeadChip

**DOI:** 10.1080/15592294.2022.2123898

**Published:** 2022-10-14

**Authors:** Sarah Holmes Watkins, Karen Ho, Christian Testa, Louise Falk, Patrice Soule, Linda V. Nguyen, Sophie FitzGibbon, Catherine Slack, Jarvis T. Chen, George Davey Smith, Immaculata De Vivo, Andrew J. Simpkin, Kate Tilling, Pamela D. Waterman, Nancy Krieger, Matthew Suderman, Caroline Relton

**Affiliations:** a MRC Integrative Epidemiology Unit, Population Health Sciences, Bristol Medical School, University of Bristol, Bristol, UK; b Bristol Bioresource Laboratories, Population Health Sciences, Bristol Medical School, University of Bristol, Bristol, UK; c Department of Social and Behavioral Sciences, Harvard T H Chan School of Public Health, Harvard University, Boston, MA, USA; d Integrative Cancer Epidemiology Programme (ICEP), Population Health Sciences, Bristol Medical School, University of Bristol, Bristol, UK; e Program in Genetic Epidemiology and Statistical Genetics, Department of Epidemiology, Harvard T.H. Chan School of Public Health, Boston, MA, USA; f Department of Medicine, Brigham and Women’s Hospital and Harvard Medical School, Boston, MA, USA; g School of Medicine, National University of Ireland Galway, Galway, Ireland

**Keywords:** DNA methylation, Illumina Infinium MethylationEPIC BeadChip, DNA input, low DNA, reliability

## Abstract

DNA methylation (DNAm) is commonly assayed using the Illumina Infinium MethylationEPIC BeadChip, but there is currently little published evidence to define the lower limits of the amount of DNA that can be used whilst preserving data quality. Such evidence is valuable for analyses utilizing precious or limited DNA sources. We used a single pooled sample of DNA in quadruplicate at three dilutions to define replicability and noise, and an independent population dataset of 328 individuals (from a community-based study including US-born non-Hispanic Black and white persons) to assess the impact of total DNA input on the quality of data generated using the Illumina Infinium MethylationEPIC BeadChip. We found that data are less reliable and more noisy as DNA input decreases to 40ng, with clear reductions in data quality; and that low DNA input is associated with a reduction in power to detect EWAS associations, requiring larger sample sizes. We conclude that DNA input as low as 40ng can be used with the Illumina Infinium MethylationEPIC BeadChip, provided quality checks and sensitivity analyses are undertaken.

## Background

Illumina Infinium MethylationEPIC BeadChips have been used extensively in epigenetic studies. Although Illumina recommend using at least 250ng of DNA on their BeadChips, there has been little published work examining the possibility of using less DNA than this. As DNA methylation (DNAm) profiling becomes more widespread, there is a need to ensure robust and reliable data can be generated from precious (e.g., clinical or historic) or limited (e.g., archaeological) biosamples. Three previous studies have assessed the effect of low levels of input DNA on the Illumina Infinium HumanMethylation450 BeadChip by generating data from multiple dilutions of the same biological samples. The first reported that correlations between genome-wide DNAm profiles remain above 0.96 for dilutions containing as little as 10ng of DNA [1]; the second reported correlations with input of 1 µg for total input as low as 10ng remained above 0.92 [2]. The third and most recent reported that input of 125–500ng total DNA resulted in highly replicable DNAm data for measurements taken over two days; and that 63ng and under total DNA input resulted in less replicable data [3]. However, no study has yet investigated the expected increase in signal variability or noise induced by low input DNA and its impact on statistical power to detect associations with DNAm; this is important because a number of studies have demonstrated that many probes on these BeadChips have low reliability, particularly where DNAm sites are either highly methylated or unmethylated and have low variance [4–6], and conceivably this might be exacerbated by low levels of input DNA applied to the BeadChip. Additionally, no comparison of data generated using different input levels has yet been carried out using a large population dataset.

Here we assess whether low yields of input DNA are sufficient to reliably detect associations with DNA methylation measured using the Illumina Infinium MethylationEPIC BeadChip. The study consists of two parts: an initial analysis, where we assess reliability and noise within a single sample at three DNA concentrations; and a subsequent assessment of total input DNA on data quality and power to detect EWAS associations, using an independent population-based DNAm dataset of 328 individuals from the My Body My Story (MBMS) study [7]. We believe this is the first study assessing the impact of low input DNA explicitly utilizing data from a large and socially diverse cohort.

## Materials and methods

### Study participants

The initial analysis (which we refer to as Study 1) included varied DNA dilutions from a single source, utilizing a DNA sample pooled from several individuals stored at −80°C. Unfortunately no data were available about the individuals contributing to this pooled sample. The sample was used to generate three dilutions resulting in three quantities of total DNA input (40ng, 200ng, and 400ng), in quadruplicate, resulting in 12 samples. Concentration of DNA was assessed using a Thermo Scientific NanoDrop™ spectrophotometer. We selected 40ng as the lowest reasonable quality based on previous work [1,2], and 200ng and 400ng as higher input comparisons.

The second analysis (which we refer to as Study 2) utilized the MBMS cohort. MBMS is a cohort recruited from four community health centres in Boston between 2008 and 2010, and was designed to investigate how racial discrimination affects risk of cardiovascular disease, taking into account a range of social and environmental factors. The cohort and recruitment procedures have previously been described in detail [7]; briefly, the study recruited 1005 individuals who met study inclusion criteria and were randomly selected from the patient rosters of the community health centres. Participants were eligible if they were aged between 35 and 64 years, were born in the US, and self-identified their race/ethnicity as white non-Hispanic or black non-Hispanic.

Among the 1005 MBMS participants, 85% provided a finger prick blood sample on to filter paper (409 black; 466 white), and consequently biological material was limited and in some instances of poor quality. Blood spots were stored at −20°C, and DNA was extracted from blood spots using the QIAamp DNA Investigator Kit for FTA and Guthrie cards, with samples randomized across 96 well plates. Of the 875 participants who provided blood spots, 472 of the samples were judged to be suitable for DNA extraction (blood spots judged not to be suitable were primarily the first community health centre where recruitment took place, whose membership was predominantly white). Of those, 48 yielded less than 40ng of DNA, the lowest input level investigated in Study 1, so we removed them from further analysis. Although 40ng showed higher variability in study 1, most probes passed quality thresholds so we included samples with inputs as low as 40ng in study 2 to maximize study sample size whilst being mindful that the samples with lower DNA input might be of reduced quality. The amount of DNA extracted was assessed using Invitrogen Quant-iT™ PicoGreen™ (Thermo Fisher Scientific). After removing a further 96 participants from the sample set due to poor quality DNA extraction (as determined by high numbers of undetected probes on the EPIC BeadChip), there were 328 participants with DNA methylation data for analysis. DNAm data were generated using the Illumina Infinium MethylationEPIC BeadChip as described below.

### DNA methylation data generation

For both studies, extracted DNA was bisulphite converted with the EZ DNA Methylation-Lightning™ Kit (Zymo Research) according to the manufacturer’s instructions. The eluant from the bisulphite-converted DNA was then applied to the Illumina Infinium MethylationEPIC Beadchip to measure DNA methylation, according to the manufacturer’s protocol. The EPIC BeadChips were scanned using Illumina iScan, with an initial quality review conducted with GenomeStudio. Sample QC and normalization were conducted using the pipeline implemented in the *meffil* R package, which has previously been described in detail [8]. Blood cell composition was estimated for MBMS using a deconvolution algorithm [9] implemented in *meffil*, based on the ‘blood gse35069 complete’ cell type reference. DNA methylation is reported in beta values; this measures methylation on a scale of 0 (0% methylation) to 1 (100% methylation).

### Study 1: assessing reliability of DNAm measurement with low input DNA

Scripts to conduct all analyses can be found at https://github.com/shwatkins/Low_input_DNA. Using the single pooled sample of DNA described above, we used two methods to assess the reliability of DNAm measurements at different input DNA levels. Firstly, we assessed how well the measurements at the lower input levels (200ng and 40ng) replicate the measurements obtained with 400ng input DNA. To do this we calculated the mean methylation at each DNAm site across the four technical replicates at each input level. We then partitioned DNAm sites into bands based on their methylation level measured at 400ng (used as the reference level) in increments of 5%. Within each partition we calculated the standard deviation of the DNA methylation levels across all sites in the partition and visualized this variation using boxplots at 40ng and 200ng. Stronger replication of the 400ng measurements would correspond to smaller variation within each partition.

Secondly, we assessed the noise in DNAm measurement within each of the three DNA input levels using their four replicates. At each DNAm site, we took the mean of replicates 1 and 2, and used these means to partition the dataset into bands of 5% methylation as we did for the first analysis. Within each partition we then calculated the mean of replicates 3 and 4 at each DNAm site. We visualized the variation within each partition using boxplots of the mean of replicates 3 and 4 for all sites within the partition. Levene’s test (*leveneTest* in the R package car) was used to determine whether lower DNA input was associated with greater variance within each partition. Greater measurement noise would correspond to greater variance. As we tested 20 partitions, we used a p-value threshold corrected for multiple tests (p < 0.05/20).

### Study 2: assessing the impact of low input DNA in a cohort study

We then assessed how low DNA input affects the quality of Illumina Infinium MethylationEPIC Beadchip data using data from our cohort study, MBMS. We conducted two sets of analyses: we calculated a variety of QC-related metrics, and evaluated the effect of input DNA level on robust associations that have been reported in the DNAm literature.

We utilized two standard QC metrics to represent data quality: proportion of probes with low signal, and median methylated signal across all probes on the BeadChip. Low signal was assessed using detection p-values, which indicates confidence that the signal from a probe is detectable above background noise. We used a detection p-value threshold of 0.01 to distinguish between detection success and failure. We plotted the relationship between the number of undetected probes and DNA input level and correlated the two variables using Spearman correlation to test the strength of the association. Median methylated signal refers to the strength of probe signal due to binding of methylated DNA to a probe. We plotted median methylated signal per sample against DNA input level, and tested their association.

In addition to these QC steps, we compared DNAm measurements for each sample against a gold standard derived from all 135 samples with DNA input >200ng by simply calculating the mean for each individual probe on the BeadChip across the 135 samples. For all remaining samples with DNA <200ng (n = 193), we calculated the difference between the methylation value at each probe and that of the gold standard, and summarized these differences per sample by taking the mean absolute difference, or MAD. We then evaluated the association between MAD and DNA input level using plots and by calculating Spearman correlation.

We tested whether variance in DNAm is associated with DNA input level at each site on the BeadChip using a procedure detailed elsewhere [10]. We firstly use the function *rq* (a least absolute deviation regression) from the R package *quantreg* to test the association between methylation at each cpg site and DNA input level, including batch, cell counts, age, gender, smoking, and BMI as covariates in the model. From this model we take the absolute values of the residuals, and then test for an association between those residuals and DNA input level using linear regression (*lm* in R). We extracted coefficients and p-values from the model and applied a Bonferroni-corrected threshold of 5.8e-08 (0.05/857774) to identify associated sites. We took the -log10 of the p-values and created a Manhattan plot.

To assess how DNA input level might affect the ability to detect effects in EWAS analyses, we conducted power analyses to show how power and required sample sizes differ by level of input DNA. Firstly, we performed a power analysis based on four partitions of MBMS defined by DNA input quartiles (each containing 73 to 74 individuals). Power was calculated for testing DNA methylation differences using a two-sided t-test between two groups. For each power analysis, the inputs were the same except for the probe standard deviation (sample size n was the number of participants in the quartile (n = 73 to 74)), the significance threshold was 0.05 Bonferroni-corrected for testing each of 850 K sites on the EPIC array, and the delta values (effect sizes) ranged from values as high as 0.2 which have been observed in smoking studies [11, 12] down to values as low as 0.01 that have been observed in some studies of social adversity [13,14]. Probe standard deviation differed between MBMS partition, with higher values observed in partitions with lower DNA input. We used the 90th percentile of probe standard deviation calculated in the corresponding partition of MBMS. Secondly, we ran power calculations asking instead the sample size that would be needed for power of 0.8, at a range of effect sizes, and given the SDs that we found for the quartiles of MBMS based on DNA input These analyses indicate how power varies in MBMS by DNA input level.

## Results

### Participant characteristics

Three quarters (74%) of participants in our study identified their race/ethnicity as Black non-Hispanic, 56% lived in areas with high numbers of individuals below the poverty line, and two thirds (66%) had less than 4 years of college education. Characteristics of the 328 participants are summarized in Table 1. DNA quantity is marginally associated with smoking status (lower quantities for former and never smokers compared to current smokers), race/ethnicity (lower quantities for white participants), and education (with less than high school education as the reference group; lowest quantities for participants with <4 years of college education, and highest quantities for participants with less than high school education).

**Table 1.** Characteristics of the 328 MBMS participants with DNAm data passing QC.

		N (%) unless otherwise stated (total = 328)	Regression coefficient/ Mean input DNA (ng)	Association with total input DNA (p value)
Age	Mean (years)	48.9 (mean)	0.05	0.97
	Standard deviation (years)	7.9 (SD)		
Gender	Women (cis-gender)	210 (64%)	231.7 ng	reference
	Men (cis-gender)	118 (36%)	201.1 ng	0.13
Smoking	Current	150 (46%	248.7 ng	reference
	Former	63 (19%)	189.9 ng	0.03
	Never	115 (35%)	201 ng	0.03
Race/ethnicity	Black non-Hispanic	242 (74%)	234.2 ng	reference
	White non-Hispanic	86 (26%)	182.7 ng	0.02
Census tract poverty, % (2005–2009)	<5% below poverty line	17 (5%)	230.2 ng	reference
	≥5%,<10% below poverty line	53 (16%)	246.1 ng	0.75
	≥10%,<20% below poverty line	75 (23%)	176.4 ng	0.26
	≥20%,<40% below poverty line (‘poverty area’)	131 (40%)	231.7 ng	0.97
	≥40% below poverty line (‘extreme poverty area’)	52 (16%)	227.7 ng	0.96
Education	Less than high school	42 (13%)	273.7 ng	0.003
	> High school, < 4 years college	218 (66%)	226.2 ng	0.02
	4+ years college	68 (21%)	170.3 ng	reference

### DNA methylation data

**Figure 1.**
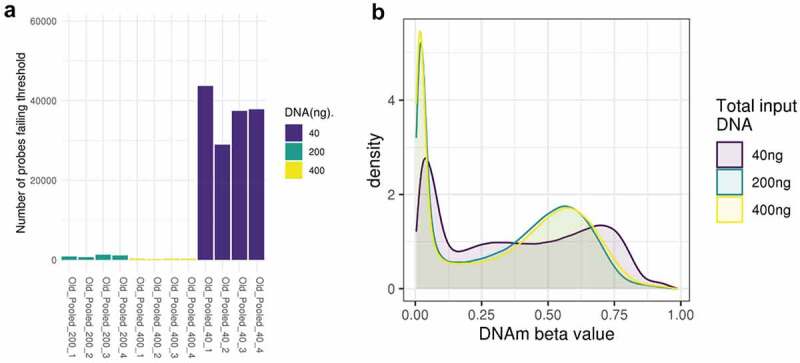
A: Number of probes that fail the detection p-value at 40ng, 200ng and 400ng. Each bar represents one sample. B: Density of methylation beta values across the EPIC BeadChip for 40ng, 200ng and 400ng DNA (post normalization).

In Study 1 quality control identified 58,072 probes for removal, including 55,706 that failed detection at the standard threshold of 0.1 (predominantly in samples with 40ng input DNA – see Figure 1a). Probes failing detection in Study 1 had 3.4% lower GC content (t -test p value<2.2e-16), suggesting that lower GC content might contribute to weaker probe binding where DNA quantities are low. There was little evidence for a difference in SNP frequency between the excluded and included probes (chi squared test p = 0.09). This left 807,787 CpG sites for further analysis. For MBMS (Study 2) we generated DNAm data for the 424 participants with over 40ng of DNA. We removed 96 participants because the blood spot samples were suspected to be poor quality and had problematic extraction; this was confirmed by very high numbers of undetected probes (up to 20%) on the EPIC BeadChip. These 96 samples had substantially lower levels of DNA than the 328 remaining for analysis (136.8ng vs 220.7ng; t-test p = 1.9e09); due to the problems with extraction they were not included in the analyses. Quality control identified 8,085 probes for removal, including 8,018 probes failing detection, leaving a total of 857,774 DNAm sites for analysis. Probes failing detection in Study 2 had 5.4% lower GC content (t -test p value<2.2e-16), suggesting again that lower GC content might contribute to weaker probe binding where DNA quantities are low. Additionally, there was evidence of a higher proportion of SNP frequency in the excluded probes in Study 2 (chi squared test p = 1e-05). Of the 328 participants, 35 samples had a mismatch between the gender they reported in the study and sex as predicted by probe signal intensities targeting sites on the X and Y chromosomes. Furthermore, whereas correlation between chronological age and age estimated from DNA methylation was reasonable for the age range in MBMS (SD 7.9) in the 293 participants who did not have a mismatch (Horvath clock R = 0.63, Hannum clock R = 0.69), correlation among the 35 with a mismatch was very low (Horvath clock R = −0.01, Hannum clock R = 0.18). We included these 35 samples in our assessment of data quality using QC analyses as they displayed no evidence of low quality, and there was no relationship between predicted sex/gender mismatch and DNA concentration (p = 0.72, Wilcoxon rank sum test); but they were removed from the power analysis, leaving 293 individuals in the power analysis.

### Study 1 results: assessing reliability of DNAm measurement with low input DNA

**Figure 2.**
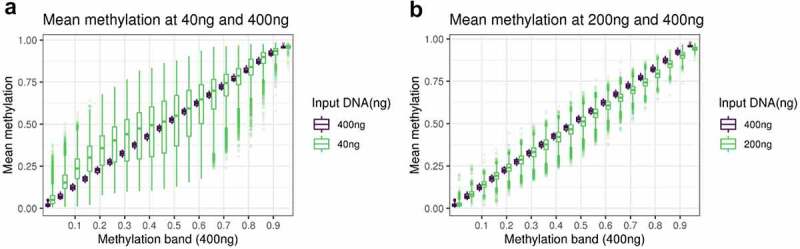
A: boxplot of the methylation of DNAm sites at 40ng, grouped in bins of 0.05 based on the methylation level of the DNAm site at 400ng. B: boxplot of the methylation of DNAm sites at 200ng, grouped in bins of 0.05 based on the methylation level of the DNAm site at 400ng.

The overall distributions of the methylation measurements across the BeadChip are virtually identical at 200ng and 400ng of input DNA, but it is skewed towards higher methylation levels for the 40ng dilution (Figure 1b). To investigate the reliability of methylation measurements when samples have low input DNA, we assessed how well measurements at 200ng and 40ng replicated those at 400ng by binning methylation sites according to methylation levels determined at 400ng, our reference. For both 40ng and 200ng, variance within each bin tends to be larger in bins representing intermediate methylation levels at 400ng. The main difference is the variation as measured by standard deviation tends to be 2–4 times larger at 40ng (SD = 0.02 to 0.17) than at 200ng (SD = 0.01 to 0.04) (Figure 2b,c; Supplementary Table S1). This indicates a reduced replication of 400ng signal at 40ng compared to 200ng.

**Figure 3.**
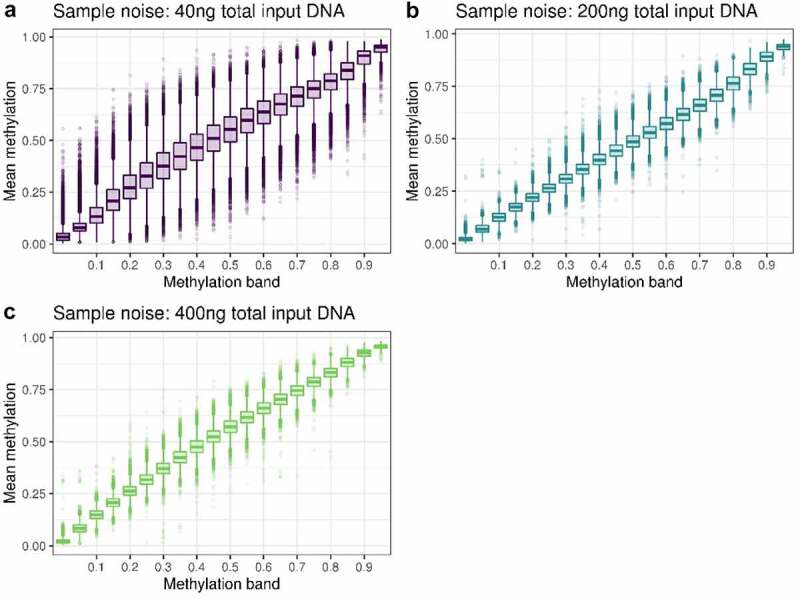
Plots of sample noise at **A** 40ng, **B** 200ng and **C** 400ng total input DNA. All CpG sites were binned into 5% partitions of methylation beta value based on the mean of replicates 1 and 2, and the mean of replicates 3 and 4 was used to create the boxplots.

As we had quadruplicate measurements for the three DNA input levels we were also able to assess the noise within each input level. This is important because measurements by Illumina Infinium MethylationEPIC Beadchips are known to be noisy, and low concentrations of DNA may exacerbate this issue [6,15]. To assess noise at each DNA input level, we used two replicates to partition methylation sites by methylation level, and then calculated the variance of each bin from the other two replicates. Plotting these bins (Figure 3) suggests 40ng results in increased within-sample noise. Using Levene’s test of variance to compare these bin variances between DNA input levels, we show that 200ng is noisier than 400ng in 17 out of the 20 partitions (at p < 0.05/20); and that 40ng is noisier than both 400ng and 200ng in all 20 partitions (at p < 0.05/20). This demonstrates that as DNA input level decreases, measurement noise increases. Levene’s test statistics are detailed in Supplementary table S2.

### Study 2 results: assessing the impact of low input DNA in a cohort study

**Figure 4.**
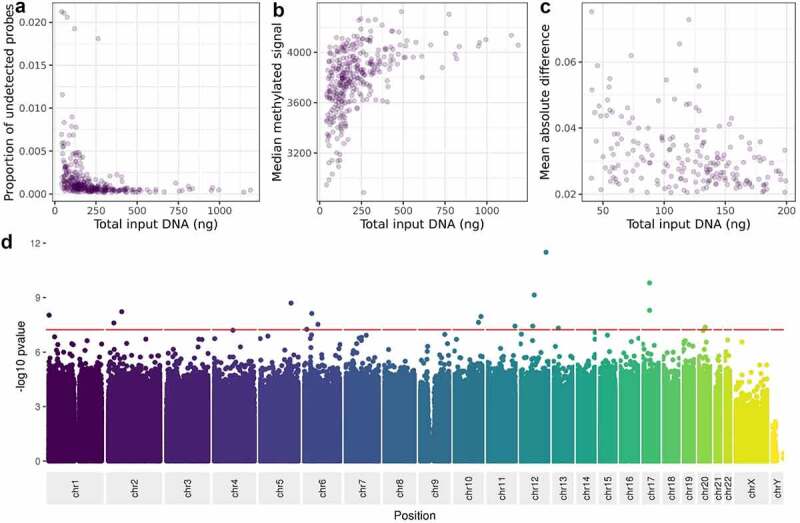
The relationship between DNA input level and, A: proportion of probes failing detection p-value, B: median methylated signal, C: mean absolute deviation from a composite of the high-input samples, D: variance at each site on the Illumina Infinium MethylationEPIC Beadchip.

DNA for the MBMS cohort was extracted from dried blood spots and resulted in a range of DNA quantities for the 472 participants for whom DNA was extracted (mean 173ng, range 0ng to 1186.8ng). As we excluded participants with less than 40ng DNA, and those with poor quality DNA extraction, DNA quantities were higher for the 328 participants who were included in the analyses included in this paper (mean 220.7ng, range 40.6ng to 1186.8ng). We assessed the impact of DNA input level on the quality of the MBMS DNAm data in three ways: the proportion of probes failing detection p-value, the median strength of methylated signal, and the mean absolute deviation of samples with lower than recommended DNA (200ng) in comparison to a gold standard based on measurements from the samples with at least 200ng DNA. For samples passing undetected probe QC checks, the proportion of undetected probes increases strongly as input DNA decreases (Spearman’s rho = −0.61,p = <2.2e-16) (Figure 4a). Similarly, although median methylated signal is correlated with DNA input level (Spearman’s rho = 0.55, p < 2.2e-16), the signal also does not fall below the threshold used in typical Illumina QC pipelines (3 standard deviations from the mean [8]; (Figure 4b). Finally, as expected, samples with lower DNA input level tend to have higher mean absolute deviation from the gold standard based on samples with at least 200ng of DNA (Spearman’s rho = −0.37, p = 1.1e-08; Figure 4c). Samples with lower DNA input (<114ng) also have higher probe standard deviation (90^th^ percentile SD = 0.76) than those with higher DNA input (>270ng; 90^th^ percentile SD = 0.068), showing that low input is associated with higher measurement variation. Thus, we have shown that although samples with as little as 40ng can pass standard QC thresholds, measurement quality and precision decrease with input DNA levels.

We then asked whether low DNA input level affects the variance of methylation measurements at specific individual sites on the BeadChip. Using linear regression with a BeadChip-wide Bonferroni-corrected threshold of 5.8e-08, we observe associations between variance in methylation value and DNA input level at 17 sites (Figure 4d and Supplementary table S3). These sites are enriched for lower GC content only for B allele probes (used as part of type I probes; t-test p value = 0.02). However, they are not enriched for coincident common SNPs (chi squared test p = 0.26) or cross reactivity (chi squared test p = 1).

**Figure 5.**
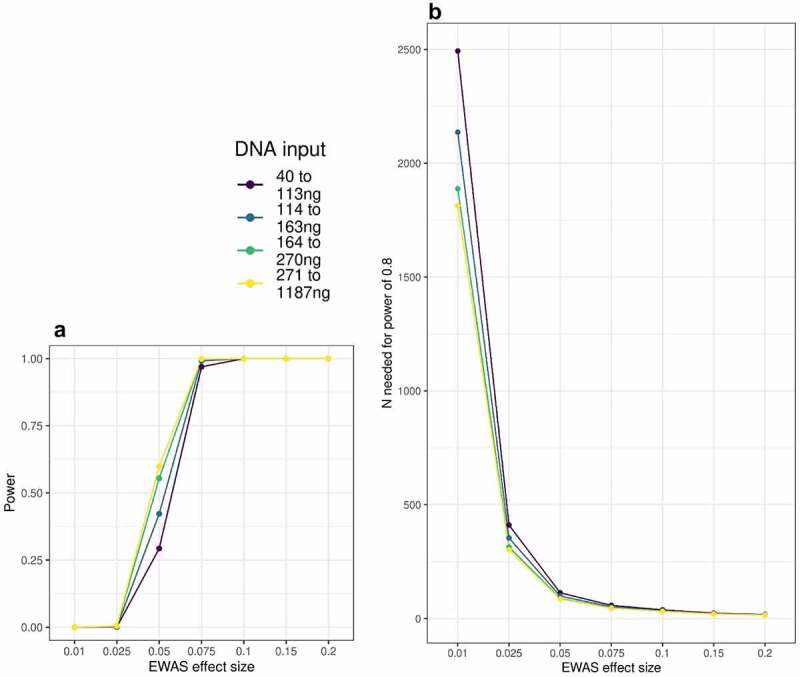
**A**: Power to detect EWAS associations in MBMS quartiles based on DNA input levels, at a range of effect sizes. **B**: Number of participants needed to detect EWAS effects at 80% power, based on DNA input level.

Finally, we asked to what extent DNA input level might affect power to detect associations in EWAS analyses. Using MBMS, we find that power is reduced for lower DNA input levels. For example, power to detect a 5% DNA methylation difference in n ~ 70 samples decreases from <0.5 down to >0.3 when decreasing input DNA from above 270ng to below 114ng (Figure 5a). DNA methylation differences below 5% are commonly observed in published EWAS; in the EWAS catalogue [16] (as downloaded on 06/05/2022), 65% of CpG-phenotype associations at p < 2.4e-07 have differences of less than 5%. To observe these effects with 80% power, sample sizes must be increased by at least 30% for input DNA below 114ng compared to input DNA above 270ng (Figure 5b; Table 2).

**Table 2.** Number of participants required to achieve 80% power to detect a range of EWAS effect sizes within DNA input quartiles.

EWAS effect size	40 to 113ng	114 to 163ng	164 to 270ng	271 to 1187ng
0.2	17.8	16.7	15.8	15.6
0.15	23.5	21.7	20.4	20
0.1	38.2	34.5	31.9	31
0.075	57.9	51.5	47	45.6
0.05	113.4	99.1	89.1	86.1
0.025	411.1	354.1	314.3	302.2
0.01	2492.9	2136.4	1888.3	1812.6

## Discussion and conclusions

This study demonstrates that although as little as 40ng can be sufficient to produce Illumina Infinium MethylationEPIC Beadchip DNAm data that pass standard QC checks, data quality and reliability diminish as DNA input decreases; and increased numbers of samples with low DNA may fail standard QC thresholds. However, this reduction in data quality may reduce power to detect EWAS effects. We hope this demonstration can empower studies to conduct DNAm investigations where it might have previously been assumed that samples were too limited to provide sufficient DNA; but due to the increase in both noise and variance that we have demonstrated, we would recommend caution and use of sensitivity analyses when working with less than 200ng DNA on the Illumina Infinium MethylationEPIC Beadchip.

Our evaluation of DNA from a single source at three dilutions illustrates that using 40ng of DNA produces noisier measurements than using 200ng, and using 200ng is noisier than 400ng. This corresponds to reduced agreement we report between measurements at 40ng than at 200ng compared to those at 400ng. Analysis of data from a cohort of 328 individuals shows a clear impact of decreasing DNA input on the proportion of probes failing detection and on the strength of methylation signal; this is presumably because there is less DNA binding to probes. This also appears to be the reason for the clear impact of decreasing DNA input level on increasing deviation from a gold standard composite profile based on samples with at least 200ng DNA. Importantly, our analyses show how fast data quality decreases as input DNA decreases, so our findings can be used to identify thresholds on input DNA suited to specific research questions. It is notable that data quality is acceptable as assessed by common quality control metrics when input DNA as low as 40 ng, although we did find that lower DNA input is likely to lead to an increase in probes failing quality control. If these probes were included in our analyses then it is possible lower DNA input would perform less favourably; but we removed them as future studies would be unlikely to retain probes failing QC.

We would strongly recommend that researchers using DNA input of less than 200ng should run quality checks and sensitivity analyses with the lower concentration samples. As we show DNA input is strongly associated with variance at many specific DNAm sites, we would suggest extra caution around these sites as they may be particularly affected by low DNA concentrations. We have provided the full summary statistics from this variance EWAS in Supplementary table 3 so that researchers can utilize these results with p-value or effect thresholds appropriate to their data and research question. Finally, we show that power to detect EWAS effects in a large community-based sample is reduced for a range of effect sizes for lower DNA input levels.

Strengths of our study include complementary analyses of both control and human cohort DNA samples; and both the large number and social diversity of individuals in the cohort analysis (n = 328). Social diversity is very important for study generalizability because DNAm is affected by our social environment. The main limitation is that the impact of DNA input level may well be different for differing sample types and provenances, as DNA quality is affected by storage and extraction methods. Indeed this is demonstrated by the much larger number of probes failing detection p-value thresholds from the 40ng samples of pooled frozen DNA from study 1, in comparison to samples with close to 40ng that were extracted from dried blood spots as part of the MBMS study (study 2). In some studies, including low-input samples could improve power whereas, in other studies, the noise introduced by low-input samples could actually reduce power. We therefore recommend sensitivity analyses in studies with low-input samples to determine their effects on study findings. However, we were not able to measure DNA quality in this study so cannot comment further on how this may impact results. As MBMS comprised only blood samples, there may also be differences between different tissue types. Variability in DNAm may of course differ across cohorts, as DNAm is affected by many aspects of our environment. Consequently, the effects of low input DNA in different studies will likely differ between studies, and we recommend sensitivity analyses in studies with low-input samples to determine their effects on study findings. Additionally, we did not assay less than 40ng DNA, so we cannot comment on how data quality might be affected by lower levels of DNA input; future studies may want to investigate data quality using lower inputs.

## Supplementary Material

Supplemental Material

## Data Availability

Due to the nature of this research, participants of this study did not agree for their data to be shared publicly, so supporting data is not available. Supplementary table 3 can be found on zenodo.org, DOI = 10.5281/zenodo.7006990, URL = https://zenodo.org/record/7006990#.YySK9HbMKUk
